# Aberrant Regulation of Notch3 Signaling Pathway in Polycystic Kidney Disease

**DOI:** 10.1038/s41598-018-21132-3

**Published:** 2018-02-20

**Authors:** Jessica Idowu, Trisha Home, Nisha Patel, Brenda Magenheimer, Pamela V. Tran, Robin L. Maser, Christopher J. Ward, James P. Calvet, Darren P. Wallace, Madhulika Sharma

**Affiliations:** 1 Department of Internal Medicine, University of Kansas Medical Center, Kansas City, Kansas United States; 2 Department of Biochemistry and Molecular Biology, University of Kansas Medical Center, Kansas City, Kansas United States; 3 Department of Anatomy and Cell Biology, University of Kansas Medical Center, Kansas City, Kansas United States; 4 Department of Clinical Laboratory Sciences, University of Kansas Medical Center, Kansas City, Kansas United States; 5 The Jared Grantham Kidney Institute, University of Kansas Medical Center, Kansas City, Kansas United States

## Abstract

Polycystic kidney disease (PKD) is a genetic disorder characterized by fluid-filled cysts in the kidney and liver that ultimately leads to end-stage renal disease. Currently there is no globally approved therapy for PKD. The Notch signaling pathway regulates cellular processes such as proliferation and de-differentiation, which are cellular hallmarks of PKD. Thus we hypothesized that the Notch pathway plays a critical role in PKD. Evaluation of protein expression of Notch signaling components in kidneys of Autosomal Recessive PKD (ARPKD) and Autosomal Dominant PKD (ADPKD) mouse models and of ADPKD patients revealed that Notch pathway members, particularly Notch3, were consistently upregulated or activated in cyst-lining epithelial cells. Notch3 expression correlated with rapidly growing cysts and co-localized with the proliferation marker, PCNA. Importantly, Notch inhibition significantly decreased forskolin-induced Notch3 activation and proliferation of primary human ADPKD cells, and significantly reduced cyst formation and growth of human ADPKD cells cultured in collagen gels. Thus our data indicate that Notch3 is aberrantly activated and facilitates epithelial cell proliferation in PKD, and that inhibition of Notch signaling may prevent cyst formation and growth.

## Introduction

Polycystic kidney disease (PKD) is one of the most common life-threatening genetic diseases, affecting an estimated 12.5 million people worldwide. PKD is characterized by the continuous growth of renal fluid-filled cysts that is driven by hyper-proliferation and abnormal fluid secretion of tubular epithelial cells. There are two inherited forms of PKD. Autosomal Dominant Polycystic Kidney Disease (ADPKD) is caused by mutations in *PKD1* or *PKD2*, which encode polycystin1 (PC1) and polycystin2 (PC2), respectively. Patients with ADPKD develop cysts *in utero*, but manifest complications later in life and reach end-stage renal disease (ESRD) in the 5^th^ decade of life. Autosomal Recessive Polycystic Kidney Disease (ARPKD) is caused by mutation of *PKHD1*, which encodes the fibrocystin protein. Clinical manifestations include kidney and liver cysts and result in death during infancy or childhood. While ADPKD occurs in 1:400 to 1:1000 individuals ARPKD is comparatively rarer with a prevalence of 1:20,000–1:40,000^1^. The PKD proteins, PC1, PC2 and fibrocystin, localize to primary cilia. PC1 and PC2 form a complex and are thought to regulate intracellular calcium (Ca^2+^)^2–5^. *PKD1*-null renal epithelial cells and ciliated cyst-lining epithelial cells derived from ADPKD patients showed a lack of flow-induced increases in intracellular calcium^6,7^. Decreased intracellular calcium levels in ADPKD cells in combination with activation of adenylate cyclase (AC) and accumulation of cAMP via vasopressin or somatostatin receptors lead to increased cell proliferation^8,9^. Recent studies and clinical trials targeting this axis have provided encouraging results^10^. The vasopressin receptor antagonist, Tolvaptan, has shown success in clinical trials, but also causes side effects, and thus its efficacy continues to be studied^10,11^. Despite many advances, the molecular basis of this disease is poorly understood, and intracellular signaling pathways that are dysregulated in PKD may present additional potential targets of therapy.

Notch signaling is activated when Notch ligands, Delta like 1 (Dll1), Delta like2 (Dll2), Delta like 3 (Dll3), Delta like 4 (Dll4), Jagged1 (J1) or Jagged 2 (J2) bind to Notch receptors, Notch1 (N1), Notch2 (N2), Notch3 (N3) or Notch4 (N4). Binding of ligand to a receptor results in a series of proteolytic cleavage events initiated by presenilin-dependent gamma secretase-like proteases. This releases the Notch receptor intracellular domain (NICD), which translocates into the nucleus. In the nucleus, the NICD associates with a transcription factor, CSL (CBF-1, suppressor of hairless, Lag2) and activates the expression of the Notch effector protein family, Hairy enhancer of split (Hes and Hey)^12–14^. While Notch signaling is essential for nephrogenesis, suppression of Notch signaling is required for terminal differentiation, and aberrant activation of the pathway results in several kidney pathologies^15,16^. Notch signaling is upregulated in mature podocytes and tubular cells in diabetic nephropathy and many other kidney diseases^17,18^. The N1 receptor has shown to be the central molecule mediating kidney pathologies and is involved in cell proliferation, apoptosis and fibrosis. The roles of the N2, N3 and N4 receptors are not well-established and different Notch receptors appear to be activated in different disease pathologies^12,19^. Conditional inactivation of Notch signaling in S-shaped bodies during kidney development results in formation of proximal tubular cysts in mice^20,21^. However, a direct role of the Notch signaling pathway in PKD has not been shown in mammals.

In the present study, we sought to determine the expression pattern and contribution of Notch signaling pathway components in PKD, using both ARPKD and ADPKD mouse models and ADPKD patient samples. The results show that the Notch pathway, particularly N3, is increased in all PKD mouse and human samples studied. Cells expressing high N3 were of collecting duct and proximal tubule origin and were proliferative in nature. Moreover, inhibition of Notch signaling using gamma secretase inhibitors resulted in decreased proliferation and attenuation of cyst progression *in vitro*.

## Results

### Notch pathway expression in the *cpk* mouse model of ARPKD

**Figure 1:**
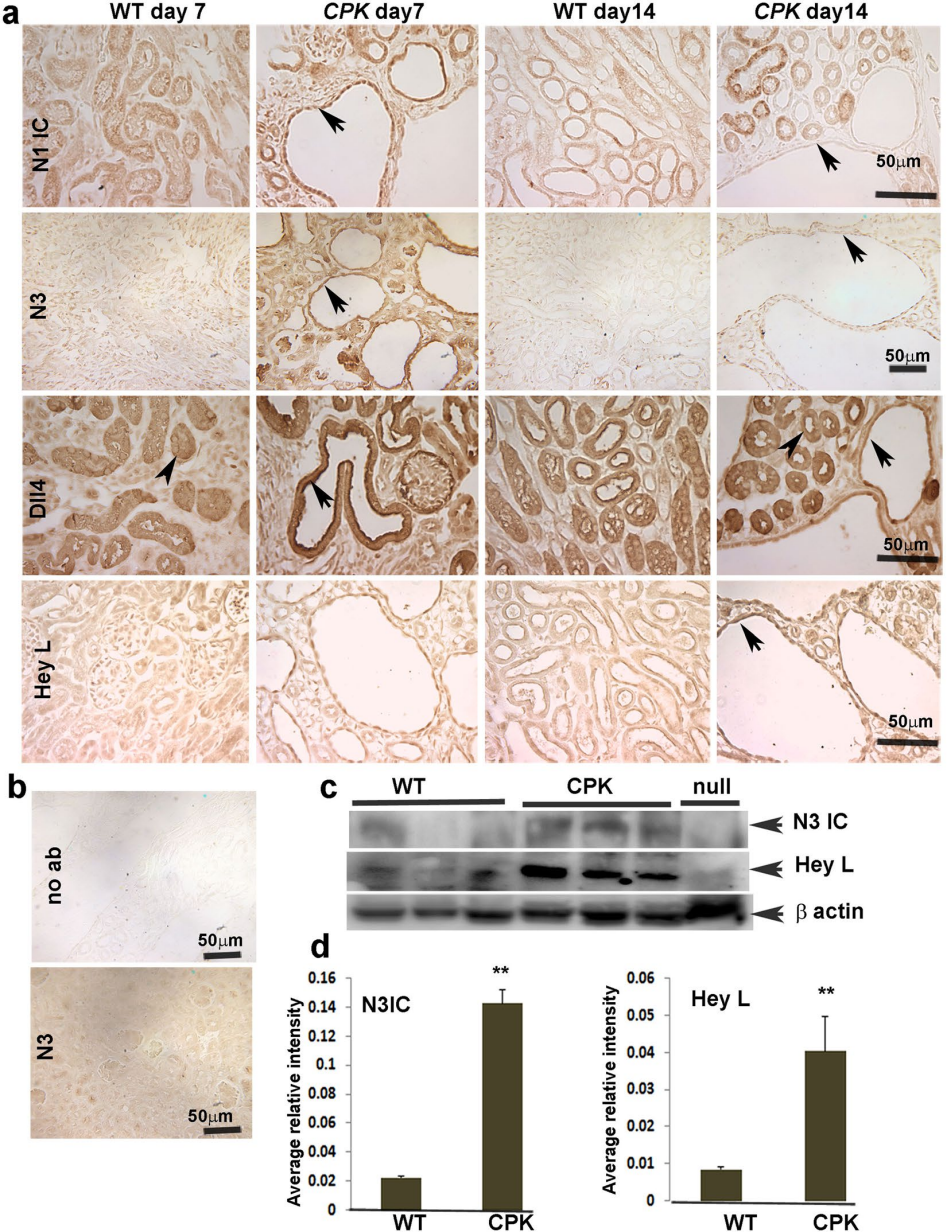
Expression pattern of Notch pathway members in kidneys of ARPKD mouse model: (**a**) Immunohistochemistry (IHC) for N1ICD (Notch1 intracellular domain), N3 (Notch3), Dll4 (Delta like 4), and Hey L was performed on paraffin sections of P7 and P14 WT and *cpk* kidneys. Arrows point to expression in cyst-lining epithelial cells. Arrowheads in third row point to non-cystic tubular cells with Dll4 expression. Images shown are representative of three independent experiments performed in duplicate. (**b**) Upper panel represents a no primary antibody control. Lower panel shows IHC for N3 on N3-null mouse kidney section to verify antibody specificity. (**c**) Western Blot for N3IC and Hey L on lysates of P15 WT and *cpk* kidneys (n = 3), and of N3-null mouse kidneys to verify antibody specificity. (**d**) Quantitation of WBs for N3IC and Hey L. **P < 0.01.

To determine whether Notch signaling is modulated in PKD, we examined protein expression of Notch pathway members in various mouse models of PKD. The *cpk* mutant is a well-studied model of ARPKD. *cpk* mice harbor a homozygous mutation in *cys1*, which encodes the cilia-associated protein, cystin, and exhibit rapidly-progressing cystic disease that leads to renal failure and death typically by 3 weeks of age^22–24^. We obtained *cpk* kidney sections at postnatal day 7 (P7) when cyst-lining cells are rapidly proliferating and at P14 when cysts have enlarged greatly and mutants are nearing end-stage renal disease (ESRD). Immunohistochemistry (IHC) was performed to localize the expression of Notch pathway members. The results revealed that the Notch1 intracellular domain (N1ICD) was upregulated in the cyst-lining epithelial cells of P7 *cpk* mutants compared to control non-cystic littermates (Fig. 1). By P14, N1ICD staining was less intense in the epithelial cells of large cysts as compared to cells lining dilated tubules of *cpk* kidneys, but staining was still more intense than in kidneys of wild type (WT) mice. N2 and N4 expression was not altered between WT and *cpk* mice at either age (Fig. S1). N3 exhibited ubiquitous expression in tubules and glomeruli of *cpk* kidneys at P7, with a striking increase in cystic epithelial cells. At P14, ubiquitous expression of N3 continued in *cpk* mice, albeit with lower intensity, but still elevated compared to controls (Fig. 1, arrows).

Among the Notch ligands, Jagged1 (J1), Jagged2 (J2), Delta like1 (Dll1), Delta like3 (Dll3) and Delta like4 (Dll4), J1 showed slightly increased expression in the cyst-lining epithelial cells of P7 and P14 *cpk* kidneys (Fig. S1). Dll4 was expressed in tubular cells of both WT and *cpk* mice (arrowheads Fig. 1), however, the expression was more intense in epithelial cells lining cysts and non-dilated tubules of P7 *cpk* kidneys. Similar to N3, elevated expression of Dll4 continued at P14 (Fig. 1; arrows). Notch target proteins Hey L showed a slight upregulation at P7 and greater upregulation at P14 in *cpk* cystic epithelium (Fig. 1). Hes1 showed a slight increase in *cpk* renal tubules at P14 (Fig. S1). To confirm antibody specificity, the immunohistochemistry protocol without use of a primary antibody was performed on *cpk* kidney sections. Staining was not observed (Fig. 1b, upper panel). Specificity for the Notch3 antibody was further evaluated by labelling N3-null mouse sections with anti-N3 antibody. Specific labelling was not observed (Fig. 1b, lower panel). Western blots were also performed using kidney lysates of P15 WT and *cpk* mice. Figure 1c and d show that the activated form of N3 (N3 IC) (~96 kDa) and Hey L (37 kDa) are significantly elevated in *cpk* lysates compared to WT, substantiating Notch activation.

### Notch pathway expression in mouse models of ADPKD

**Figure 2:**
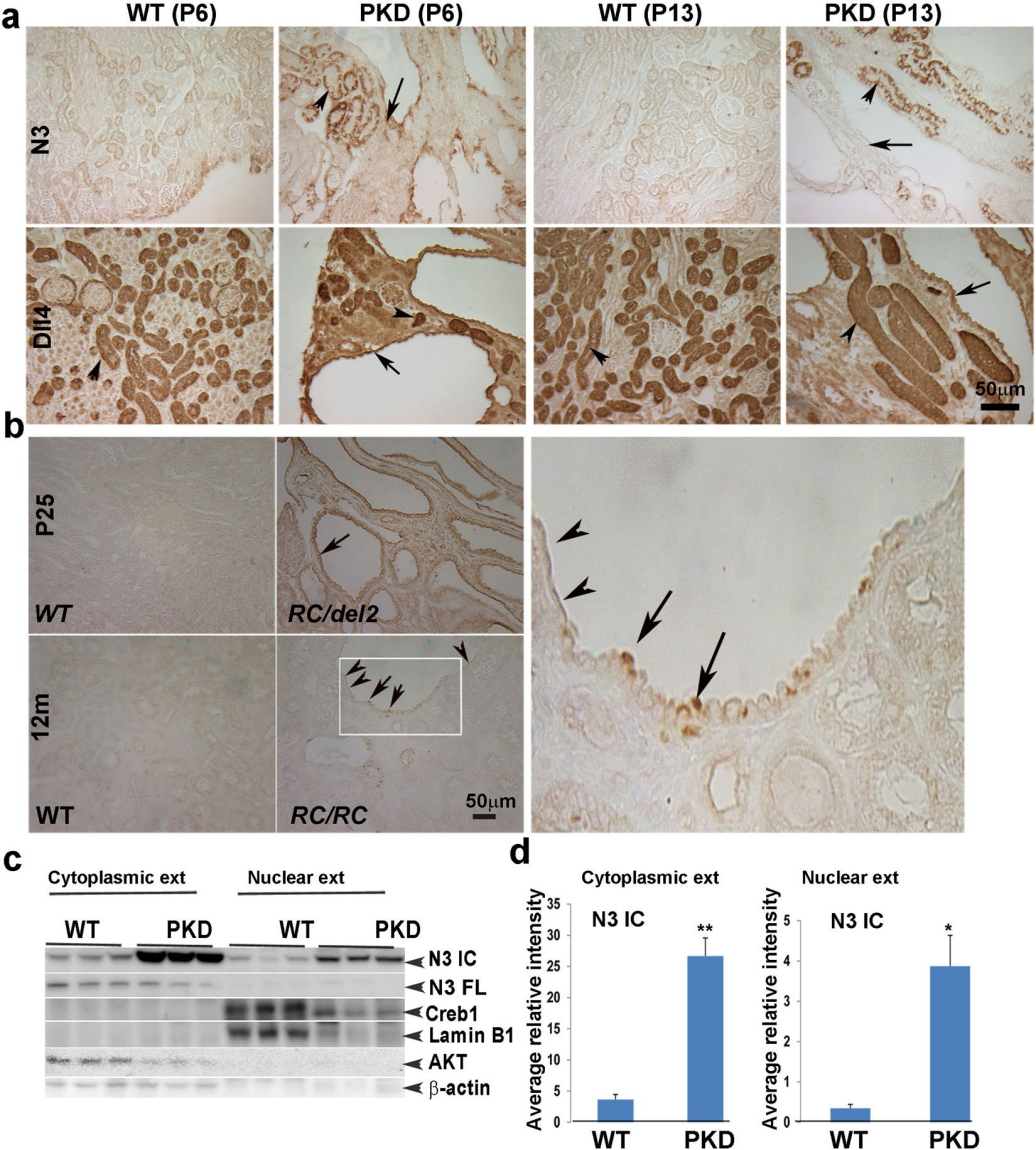
Expression pattern of Notch pathway members in kidneys of ADPKD mouse models: (**a**) IHC for N1ICD, N3 and Dll4 was performed on paraffin sections of P6 and P13 WT and *PKD1*^*CD*^ kidneys. Arrows point to high expression of N3 at P6, which reduces in cells lining large cysts at P13. Arrows also point to intense Dll4 labeling in tubules and cystic epithelia at P6, which reduces at P13. Arrowheads point to consistently high expression of Dll4 in tubules. (**b**) IHC for N3 on kidney sections of P25 *PKD1*^*RC/del2*^ (upper left panel) and 12-month-old *PKD1*^*RC/RC*^ (lower left panel) mice and WT littermates. Arrows point to cuboidal/columnar epithelial cells with N3 labeling. Arrowheads point to flattened cells with no N3 expression. Magnified view of inset shown in panel B is shown in right panel. (**c**) Western blot for N3IC and N3FL (full-length) on cytoplasmic and nuclear fractions of kidneys of P13 WT and *PKD1*^CD^ mice. Creb1 and Lamin B1 were used as nuclear markers and AKT and β-actin as cytoplasmic markers. Data for N3IC was normalized to Lamin B1 and β-actin for nuclear and cytosolic fractions, respectively. (**d**) Quantitation of WB for cytoplasmic and nuclear N3IC. *P < 0.05; **P < 0.005.

Next we studied the protein expression pattern of Notch pathway components in mouse models of ADPKD. Several ADPKD mouse models have been generated. Some are rapidly progressing, developing cysts early and rapidly leading to ESRD by P20, while others are slowly progressing, enabling mice to survive beyond one year with mild cystic disease. Both types of mouse models have been instrumental in studying the molecular biology of the disease and efficacy of pharmacological compounds. To determine Notch pathway expression in ADPKD, we used mice with collecting duct-specific deletion of the *PKD1* gene (*PKD1*^*CD*^) described previously^25^. *PKD1*^*CD*^ mice exhibit kidneys that are larger and grossly cystic in comparison to age-matched control littermates by P13. Unlike *cpk* mice, N1ICD was not expressed in P6 or P13 *PKD1*^*CD*^ kidneys (not shown). At P6, N3 immunostaining was more intense in *PKD1*^*CD*^ cystic kidneys compared to WT, displaying staining in both non-dilated and cystic tubular epithelium. At P13, N3 staining of cells lining large cysts was less intense than those lining non-dilated tubules (Fig. 2a). Dll4 was highly expressed in both WT and *PKD1*^*CD*^ mouse kidney sections. In P6 *PKD1*^*CD*^ kidneys, strikingly high expression of Dll4 was present in cells lining non-dilated tubules (Fig. 2a, arrowhead). Similar to Notch3, the expression of Dll4 reduced in cystic epithelium at P13.

Since N3 expression was consistently increased in cyst-lining cells of the rapidly progressing *cpk* and *PKD1*^*CD*^ disease models, we next examined N3 expression in other ADPKD mouse models, *PKD1*^*RC/RC*^ and *PKD1*^*RC/del2*^ ^26^. *PKD1*^*RC/RC*^ mice are slowly progressing cystic models that were generated by knock-in of a naturally occurring disease variant, *PKD1p*.*R3277C (RC)*. These mice begin to develop cysts at 3 months of age and grow progressively with age. At 12 months of age increased fibrosis is detected. The *PKD1*^*RC/del2*^ model was generated by breeding *PKD1*^*RC/*+^ animals with a *PKD1*-null model (*PKD1*^+*/del2*^) as described previously^27^. These *PKD1*^*RC/del2*^ animals show rapidly progressive disease with exponential increase in cyst size until P25 when they develop ESRD and die^26^. We used kidneys from both the *PKD1*^*RC/del2*^ and *PKD1*^*RC/RC*^ mouse models at P25 and at 12 months of age, respectively. N3 was increased in *PKD1*^*RC/del2*^ cyst-lining cells that have retained a cuboidal or columnar epithelial structure (Fig. 2b arrow). In contrast, in *PKD1*^*RC/RC*^ mutants, whose cysts progress slowly, cyst-lining cells that have flattened have no N3 expression (Fig. 2b; arrowheads), while cyst-lining cells that have retained a cuboidal/columnar epithelial structure expressed N3 (Fig. 2b; arrows, see inset from *RC/RC* in the right). This finding corroborates that N3 expression correlates with rapidly-growing cells but is lost in de-differentiated cells. We also performed IHC for N3 expression in a ciliary mouse model of PKD, the *Thm1* conditional knock-out mouse^28^, and consistently found increased expression of N3 (Fig. S2). We performed WBs on nuclear and cytoplasmic fractions of P13 *PKD1*^*CD*^ and WT kidneys. Both cytoplasmic and nuclear fractions of *PKD1*^*CD*^ kidneys showed elevated expression of N3 IC (Fig. 2c). A band observed at 224 kDa was only present in the cytoplasmic fraction and corresponds to full-length (FL) N3. The purity of nuclear extracts was verified by probing for nuclear proteins, Creb1 and LaminB1, while the purity of the cytoplasmic extracts was verified by probing blots for AKT and β-actin. A significant increase in the N3 IC expression from both nuclear (P < 0.02) and cytoplasmic extracts (P < 0.001) was observed (Fig. 2d). Together, these data suggest that N3 upregulation is a common mechanism of rapidly growing cysts.

### Notch 3 is upregulated in cystic epithelia of human ADPKD kidneys

**Figure 3:**
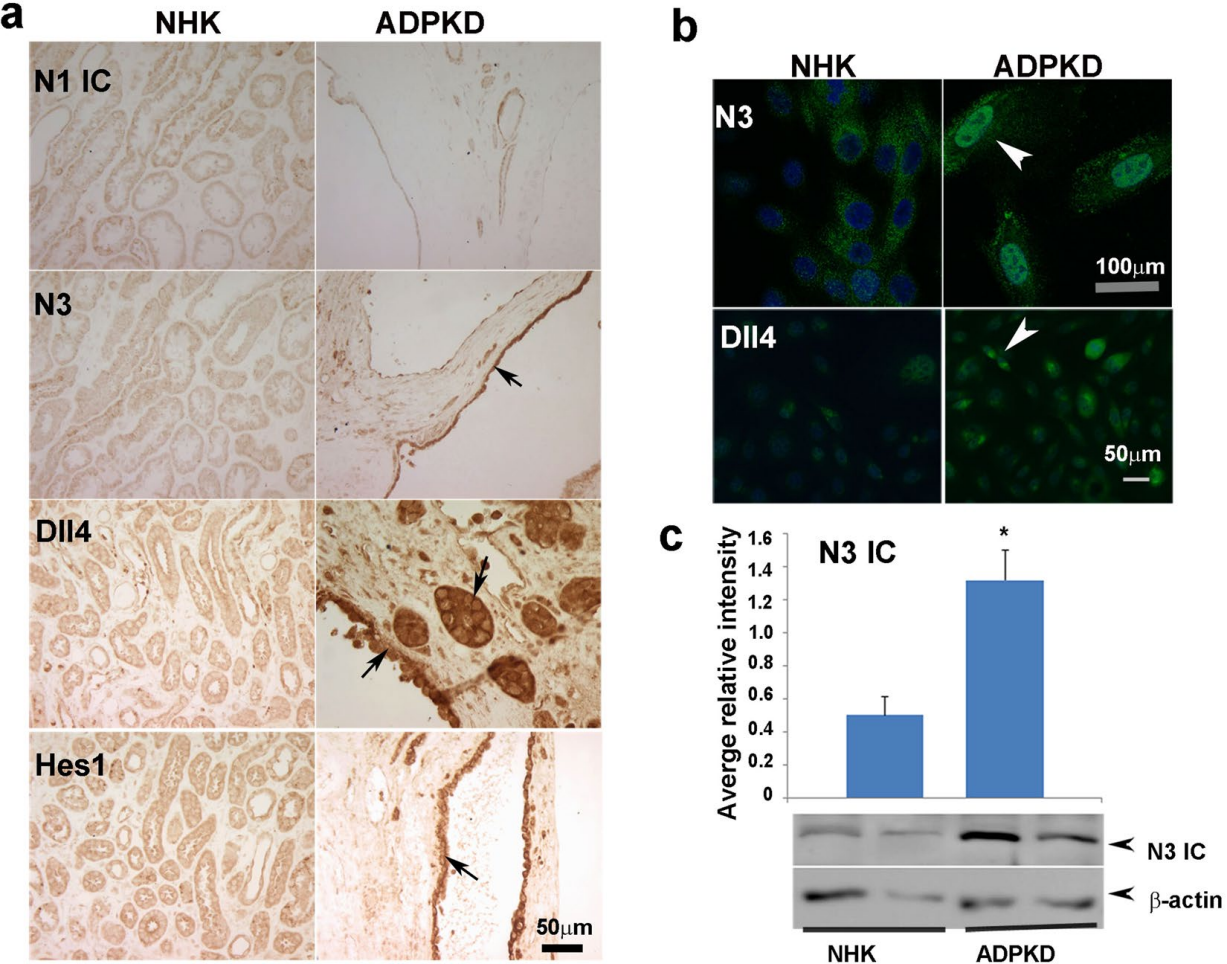
Expression pattern of Notch signaling molecules in kidneys of ADPKD patients: (**a**) IHC for N1 IC, N3, Dll4 and Hes1 was performed on NHK and ADPKD paraffin kidney sections. Arrows point to cells with high expression. (**b**) IF for N3 and Dll4 was performed on NHK and ADPKD primary renal epithelial cells. Arrow in the right bottom panel points to dividing cells with high Dll4 expression (**C**) Primary ADPKD and NHK renal epithelial cells were grown to 85–90% confluency. Western blots were performed on cell lysates to quantify N3IC levels, which were normalized to β- actin (bottom panels) (n = 6). Experiments were done at least three times in triplicate. Columns and error bars represent means ± standard errors. *P < 0.05.

To extend our findings to human ADPKD, we examined expression of Notch pathway members in kidney sections obtained from ADPKD patients and from normal human kidney (NHK) as controls. N1 ICD expression in tubules appeared similar between NHK and ADPKD sections, however the fibrotic areas in ADPKD sections had no N1ICD expression (Fig. 3a). N3 was broadly expressed in ADPKD cystic epithelium compared to NHK controls (Fig. 3a). Whereas Dll4 was minimally expressed in NHK sections, Dll4 was markedly increased in epithelia of non-dilated tubules as well as of cysts in ADPKD sections. Intense staining for the Notch target molecule, Hes1, was observed in ADPKD cystic epithelium as compared to NHK controls. Expression of N4, J1, J2, Dll1, Dll3 and Hey L proteins was not altered (Fig. S3).

Since distinguishing between nuclear and cytoplasmic labeling in tissue sections was difficult, we performed immunofluorescence for N3 on actively growing primary cells derived from cyst-lining epithelia of ADPKD kidneys and from tubular epithelia of NHK kidneys. These cells are obtained from the kidney cortex and stain positively for DBA^8^. Increased expression of N3 was seen in the nucleus of ADPKD cells showing that the protein is cleaved and represents the activated form of N3, in contrast to NHK cells in which N3 did not localize to the nucleus (Fig. 3b). We also performed Western blots to quantify N3 protein expression in these cells. The active form of N3 (N3 ICD) (~96 kDa) was significantly higher (P < 0.02) in ADPKD cells compared to the NHK controls (Fig. 3c). These studies confirmed that N3 is activated in cystic epithelial cells from human ADPKD samples.

### N3 is upregulated in proximal tubules, collecting ducts and interstitial cells of PKD kidneys

**Figure 4:**
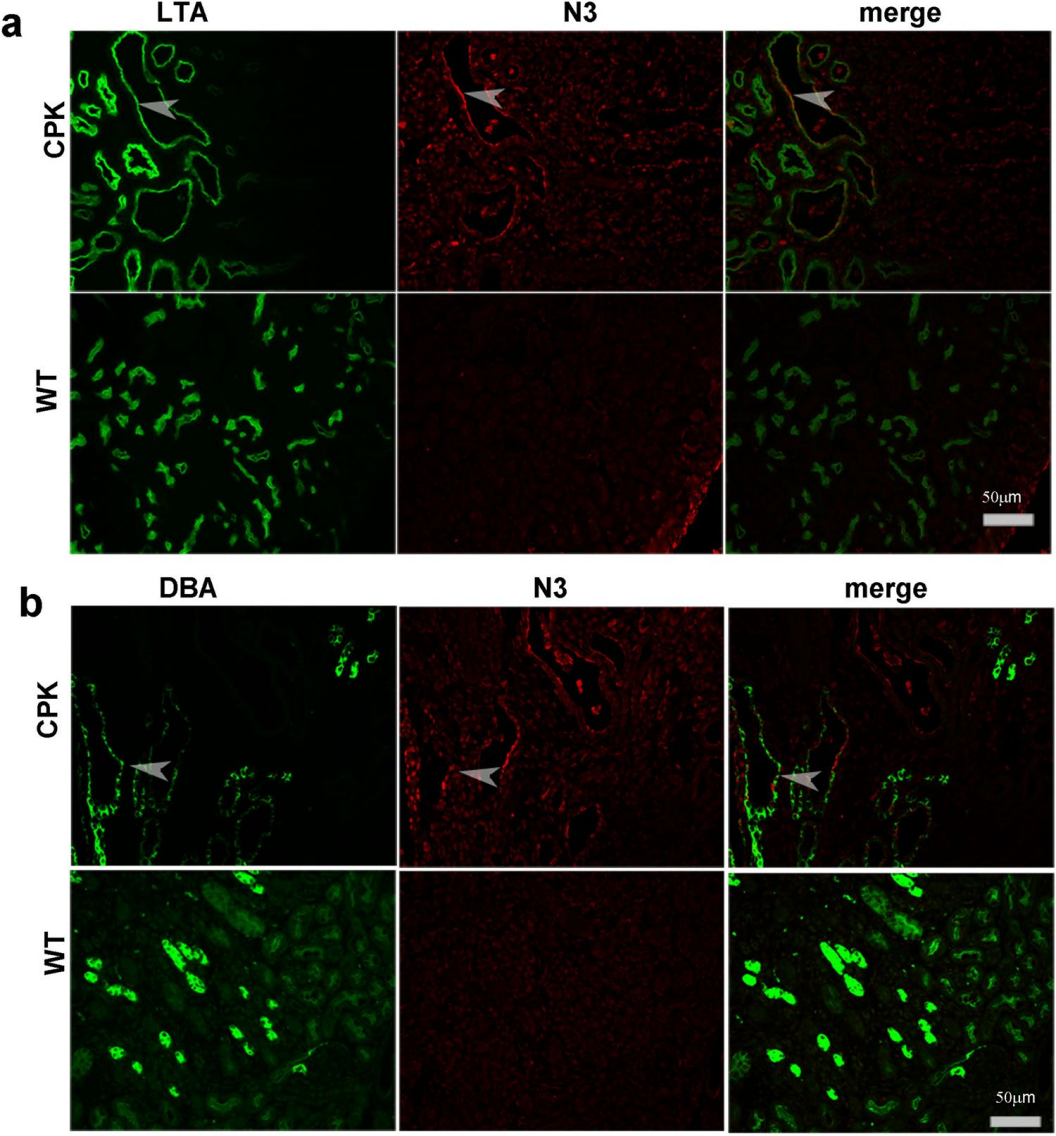
Notch3 is upregulated in proximal tubules and collecting ducts of *cpk* kidneys: (**a**) IF for N3 (red) together with incubation with fluorescein-labeled LTA was performed on paraffin sections of P7 *cpk* kidneys. (**b**) Sections were also labelled for N3 (red) and DBA (green). Co-expression of N3 with LTA or DBA is shown by arrows.

We next determined the tubular segments that express high levels of N3. We used the *cpk* mouse model of ARPKD since these mice have both proximal tubule- and collecting duct-derived cysts at P7. Co-labelling for N3 (red) and *Lotus tetragonolobus* (LTA), a marker for proximal tubules (green), was performed using immunofluorescence. *cpk* kidneys showed a higher expression of N3 in dilated tubules (Fig. 4a, upper panel,merge). These dilated tubules with high N3 expression included LTA-positive and non-LTA positive cells. In addition, interstitial cells appeared to express more N3 compared to WT (Fig. 4a, lower panel). We next labeled sections for N3 (red) and *Dolicos biflorus agglutinin* (DBA), a marker of collecting ducts (Fig. 4b, in green). We observed upregulation of N3 (in red) in cells of dilated tubules that were DBA-positive or non-DBA positive (Fig. 4b, merge). This indicates that N3 is upregulated in multiple kidney segments and cell types, including in proximal tubules, collecting ducts and interstitial cells of cystic kidneys.

### N3 expression correlates with cell proliferation and Notch inhibition reduces cyclic AMP-mediated cell proliferation in ADPKD

**Figure 5:**
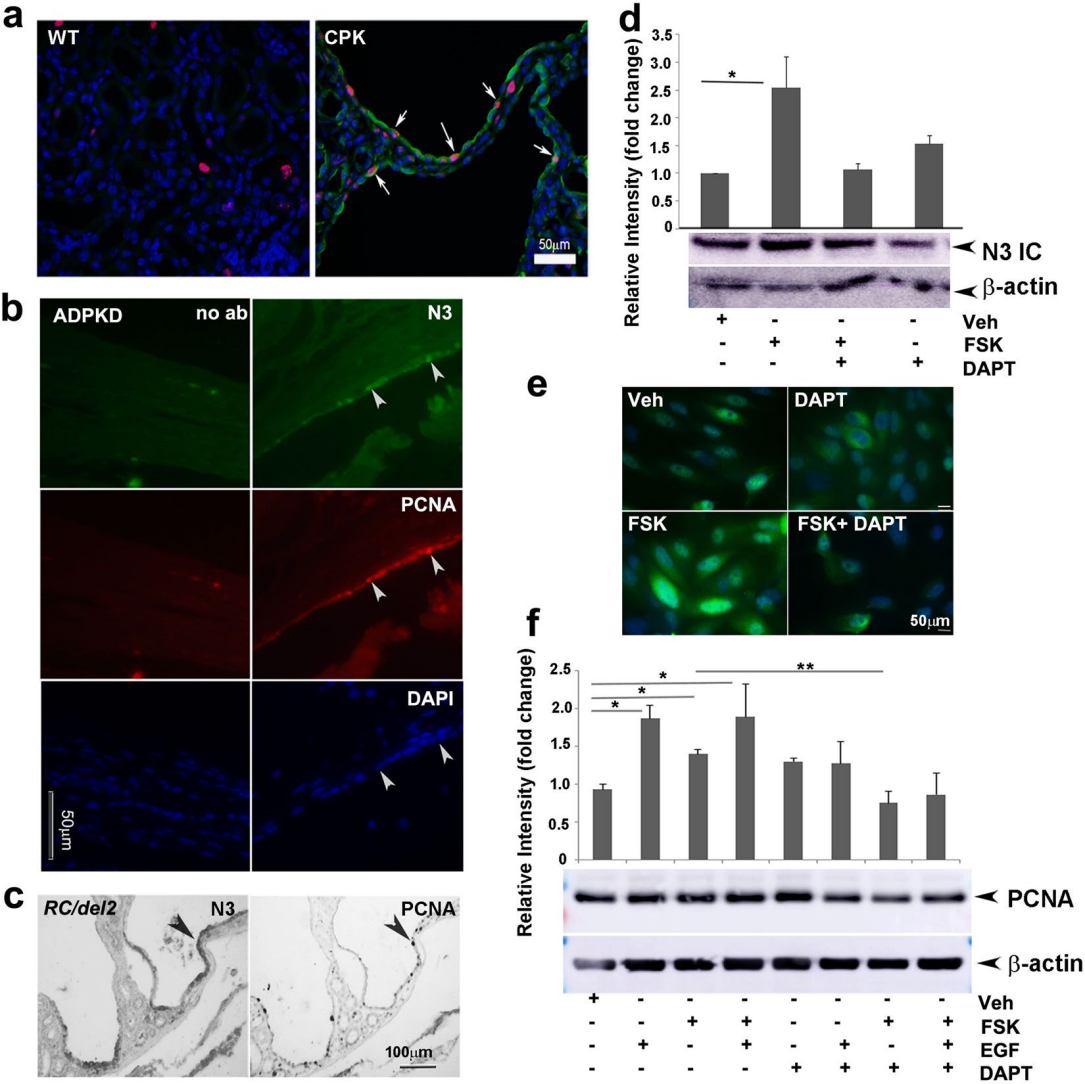
Notch3-expressing cells are proliferative: (**a**) IF for N3 (green) and PCNA (red) on paraffin sections of P14 WT and *cpk* kidneys. Nuclei were stained with DAPI (blue). (**b**) IF for N3 (green) and PCNA (red) on ADPKD kidney sections. Left panels show no primary antibody (no ab) controls. Right panels show labelling for N3 or PCNA of cyst-lining epithelial cells. (**c**) IHC for N3 and PCNA on P25 *Pkd1*^*RC/del2*^ kidney sections shows same cells express N3 and PCNA. (**d** and **e**) ADPKD cells were grown to 70% confluency, then treated with FSK or DAPT, alone or in combination for 24 hrs. N3IC expression was evaluated by Western blot analysis on cell lysates (d) or by Immunoflourescence on fixed cells (e). (**f**) ADPKD cells were treated with FSK, EGF or DAPT alone or in combination for 24 hrs. Western blot for PCNA was performed to determine proliferation profile. Values were normalized to β- actin. Experiments were performed three times using three different ADPKD cell lines. Data are represented as mean ± standard error. *P < 0.05; **P < 0.01.

Cell proliferation is an important aspect of cyst formation and growth, and our data suggest that N3 expression correlates with increased cell proliferation. Therefore, we determined whether N3 expression correlates with a marker of cell proliferation, PCNA (proliferating cell nuclear antigen). P14 *cpk* mouse kidney sections were immunostained for N3 and PCNA (Fig. 5a). PCNA-positive cells were increased in cyst-lining epithelia of *cpk* kidneys with many cells expressing both N3 and PCNA (Fig. 5a). To determine if this occurs in human ADPKD, ADPKD kidney sections were also immunostained for N3 and PCNA. Similarly, cyst-lining epithelial cells expressed both N3 and PCNA (Fig. 5b). Further, this co-expression of N3 and PCNA was shown also in cyst-lining epithelia of *PKD1*^*RC/del2*^ kidneys (Fig. 5c). This indicates that cyst-lining cells with high levels of N3 are proliferative, suggesting that N3 activation is important for cyst formation and/or expansion.

EGF and Arginine vasopressin (AVP) can stimulate cell proliferation in human ADPKD cells through activation of the MEK-ERK pathway^4^. cAMP or agonists of cAMP, such as forskolin (FSK), also induce cell proliferation of human ADPKD cells and of renal epithelia in PKD animal models via the MEK-ERK pathway^3,8^. cAMP and its agonists also stimulate chloride-dependent fluid secretion in ADPKD cells grown in collagen gels to form cysts^5^. We tested the effect of gamma secretase inhibitor, DAPT, (N-(N-(3,5-Difluorophenacetyl)-S phenylglycine t-butyl ester) (50 µM) on N3 activation. Cells were treated with FSK and DAPT either alone or together. A significant elevation of N3 IC expression (P < 0.05) was observed in FSK-treated ADPKD cells, which was reduced in the presence of DAPT (Fig. 5d). We also performed immunofluorescence for N3IC on FSK-treated ADPKD cells, and found FSK increased nuclear localization of N3IC and DAPT reduced this effect (Fig. 5e). Further treatment of ADPKD cells with FSK, EGF, and DAPT, alone or in combination for 24 hours, were carried out to evaluate the effect on cell proliferation. As expected, cell proliferation was induced in both EGF (P < 0.02) and FSK (P < 0.05) - treated cells as determined by PCNA expression (Fig. 5f). While DAPT alone did not reduce basal proliferation of ADPKD cells, the combination of DAPT with FSK reduced PCNA expression relative to FSK-induced PCNA expression of ADPKD cells (Fig. 5f; P < 0.05). This suggests that Notch pathway works through cyclic AMP-mediated cell proliferation in ADPKD. We evaluated ERK activation after 15 minutes of treatment. We found that phospho-ERK/total ERK ratios showed a decreasing trend in the presence of DAPT to FSK or FSK + EGF treatments, similar to the effect on PCNA expression (Fig. S4). This suggests that the Notch pathway may work partially through the cyclic AMP- MEK- ERK pathway to increase cell proliferation in PKD.

### Inhibition of Notch signaling can ameliorate cyst progression *in vitro*

**Figure 6:**
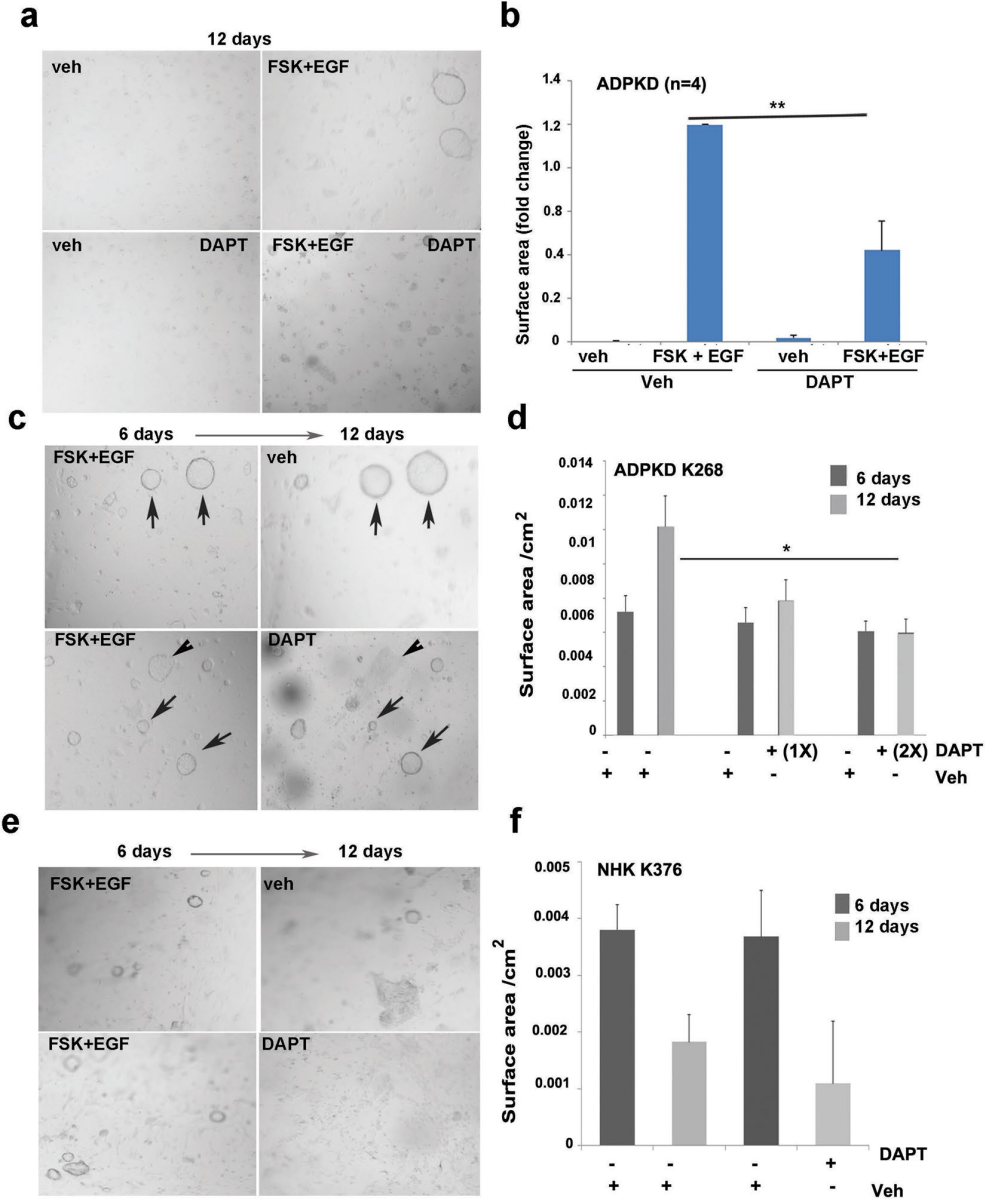
Notch inhibition suppresses cyst formation of primary human ADPKD cells: (**a**) ADPKD cells were grown in collagen gels in media with vehicle or with cyst agonists, FSK and EGF. After 6 days when cysts were formed, agonists were removed and cells were treated with vehicle or DAPT (50 μM) for six more consecutive days. Shown are representative images of cysts after 12 day-treatment. (**b**) Cyst diameters after 12-day treatment were measured and converted to surface area. Shown are differences in cyst surface area (fold change) by treatment (n = 4). Treatment with agonists in the absence of DAPT was expressed as 1.0. **P < 0.01 (**c**) ADPKD cells were allowed to form cysts in the presence of agonists for six days, then imaged (left panels). Afterward, cells were treated with vehicle or DAPT (50 μM) for another six days and the same frame was re-imaged for comparison of cyst size (right panels). Arrows in upper panel show size difference when cells were treated with vehicle alone. Arrows in lower panel show size difference in response to DAPT. Arrowhead points to a cyst that disappeared after 6-day DAPT treatment. At least 25–30 cysts were measured per treatment group. *P < 0.05 (**d**) Quantitation of cyst surface area (cm^2^) treated with vehicle or 50 μM (1x) or 100 μM (2x) DAPT. *P < 0.05. (**e**) Similarly, NHK cells were photographed after six days of agonist treatment (left panels). Agonists were removed and cells were treated with vehicle or DAPT for 6 more days. Same area was re-imaged at end of treatment (right panels). (**f**) Quantitation of NHK cyst size following vehicle or DAPT treatment. At least 25–30 cysts were measured per treatment group.

Since DAPT treatment showed an inhibitory trend in cAMP-mediated P-ERK expression and significantly reduced FSK-mediated cell proliferation, we queried whether DAPT treatment would also be effective in cells after they are induced to form 3D cysts by FSK and EGF^9^. ADPKD cells were cultured in collagen gel for 6 days in the presence of FSK and EGF to form cysts. These agonists were then removed and Notch inhibition was initiated by using DAPT (50 µm). Treatment was carried out for 6 consecutive days with either control media containing DMSO vehicle or media containing DAPT. As shown in Fig. 6a and b, DAPT alone did not influence cyst formation or growth, however in combination with FSK and EGF (agonists), decreased the surface area of FSK/EGF-induced ADPKD cysts (p < 0.01), demonstrating that Notch inhibition suppresses cell proliferation and/or chloride-dependent fluid secretion. Moreover, we followed the growth of cysts through a 12-day experiment and observed that ADPKD cysts continue to grow even after 6 days following removal of FSK and EGF agonists. However, DAPT treatment significantly halted this growth (Fig. 6c and d). Some cysts (arrowhead) even appeared to resolve, showing that DAPT may initiate cell death pathways in addition to halting cell proliferation/fluid secretion. On the other hand, while NHK cells form big cysts in the presence of EGF and FSK, unlike ADPKD cells, NHK cysts do not continue to grow after removal of the agonists, and regress in size either in the presence or absence of DAPT (Fig. 6e and f). We used two more gamma secretase inhibitors, Dibenzazepine (DBZ) and PF3084014 (2–5,7-difuoro-1,2,3,4-tetrahydronephthalen-3-ylamino)-N-(1-(2-methyl-1-(neopentylamino)propan-2-yl)-1H-imidazol-4-yl)pentanamide) in our studies. DBZ has been shown to be widely used as Notch inhibitor in basic and clinical studies^18,29–31^. PF 3084014 (PF) is currently in clinical trials for cancer and is shown to selectively inhibit gamma secretase to preferentially reduce Notch signaling^32–34^. We first show that both DBZ (2 μg/ml media) and PF (5 nm) can reduce FSK mediated N3 expression in the nucleus in ADPKD cells (S5). We also performed 3D gel cyst assays with both PF and DBZ. Similar to DAPT, ADPKD cells responded with a significant reduction in cyst volume with both the drugs in a dose dependent manner. NHK cells showed non-significant effects with DBZ but did show decrease in cyst size, probably because of the nature of the drug (S5). Taken together, these studies clearly show that FSK mediated ADPKD cells are poised to respond to Notch inhibition by gamma secretase inhibitors.

## Discussion

To our knowledge, this is the first study to evaluate Notch signaling in PKD. Our data show a specific upregulation and activation of N3 in cystic cells of multiple mouse models of PKD and in human ADPKD. N1 and J1 was upregulated in early *cpk* cysts, but not in ADPKD models. This may indicate that N1 and J1 may be differentially regulated in ARPKD and ADPKD models, and their roles need further investigation. In contrast, expression of N2 and N4 receptors and of J2, Dll1 and Dll3 ligands did not appear to be affected in PKD. Previously, we have shown that N4 is activated in many cells in HIV-associated nephropathy^19^. Recent studies have shown specific N3 activation in glomerular nephropathy^35^ N1 has been reported as the central Notch molecule mediating kidney pathologies. Thus activation of specific Notch receptors may be context-dependent and may vary with the type of renal disease and pathways misregulated in the particular disease^12,17–19^.

N3 was activated in kidneys of *cpk* mice, multiple ADPKD mouse models and ADPKD patients, suggesting that a common mechanism activating N3 exists during cyst formation in both ARPKD and ADPKD. Among ligands, Dll4 was the only one consistently increased in the PKD models. Interestingly, the expression of both Dll4 and N3 was higher in cystic epithelium of P7 *cpk* or P6 *PKD1*^*CD*^ kidneys when cysts were rapidly expanding and cells had a high proliferation rate compared to P14 or P15 mutant kidneys when cysts were very large and most tubular epithelial cells may have begun to de-differentiate. In *PKD1*^*RC/del2*^ and *PKD1*^*RC/RC*^ models, the N3 expression coincided with cystic epithelial cells that retained their normal cuboidal or columnar structure but was absent in flattened cells. Moreover, N3 expression co-localized with proliferating cystic epithelium in *cpk*, *PKD1*^*RC/del2*^ and human ADPKD kidneys, suggesting that N3 is involved in cell proliferation during cyst formation.

Cyclic AMP agonists, such as FSK, together with EGF can accelerate cyst growth by stimulating trans-epithelial Cl^-^ secretion and proliferation^36^. In ADPKD cells, cyclic AMP-dependent cell proliferation is mediated by activation of B-Raf which stimulates MEK, which in turn, activates mitogen activated protein kinase ERK, which then translocates to the nucleus to activate genes involved in cell proliferation. In cancer studies, the Notch pathway has been shown to require active signals from the ERK/MAP kinase pathway downstream of Ras^37^. Moreover EGFR protein levels differentially affected Notch signaling in many lung cancer cell lines^38^. In our study, Notch inhibition significantly reduced FSK-induced proliferation of ADPKD cells and also showed some inhibitory effects on EGF-induced proliferation. A significant reduction in FSK/EGF induced cyst size was observed when ADPKD cells were grown in a collagen gel. This shows that Notch inhibition interferes with ERK/MAPK signaling. However, whether the reduced cyst size following DAPT treatment was due only to inhibition of cell proliferation or also to increased cell apoptosis and decreased fluid accumulation is not clear.

Previous studies showed that inactivation of Notch molecules during development result in proximal tubular cysts^20,21^. Thus, we expected that there would be down-regulation of most Notch members in PKD, which was not the case. The right balance of Notch molecules may be required for proper renal cell homeostasis. N3 activation has been reported in other kidney diseases which are not associated with cysts, thus increased N3 expression by itself may not be sufficient for cyst initiation but may act in conjunction with other pathways to promote cystogenesis. Interestingly, inhibition of N3 and J1 in the *double bubble* zebrafish ciliary mutant reduced cyst formation^39–41^. Moreover, mutations in ciliary genes caused defects in Notch signaling in epidermal cells^42^. Since PKD is a ciliopathy, Notch signaling, similar to many other developmental pathways, may be involved in disruption of cilia function or vice versa in PKD^43^.

These studies implicate Notch inhibitors as potential therapies for PKD. Gamma secretase inhibitors have shown to ameliorate many kidney diseases and are in clinical trials for various cancers. In our studies, GSI’s inhibited *in vitro* cyst growth of ADPKD cells. Under normal circumstances, gamma secretases have been shown to cleave PC1 and release the C-terminal tail which translocates to the nucleus and helps maintain normal cell proliferation and apoptosis by inhibiting pro-proliferating and pro-apoptotic signaling pathways through the transcriptional co activator, p300^44^. In ADPKD cyst-lining epithelial cells, where PC1 is mutated, GSI should not cause such a mechanism but rather suppress cyst growth as a result of Notch inhibition as seen in our *in vitro* cyst assays. However, normal cells in ADPKD may also be affected^44^. Thus, targeting the specific Notch receptor appropriate to the disease type is a better option. Further studies are being designed to determine the mechanism of Notch3 activation in PKD and to target Notch3 specifically in the PKD context.

## Methods

### Animal care and protocol

*Cpk*, *PKD1*^*CD*^ and *Thm1* mutant mice were housed under pathogen-free conditions in micro-isolator cages on a high-efficiency particulate air-filtered ventilated rack. The study was carried out in accordance with the recommendations in the Guide for the Care and Use of Laboratory Animals of the National Institutes of Health. The protocol was approved by the Committee on the Ethics of Animal Experiments of the University of Kansas Medical Center (Kansas City, KS). *PKD1*^*CD*^ and *cpk* mice were anaesthetized followed by euthanasia at P6, P7 and P13 or P15 to harvest kidneys. Gene deletion in *Thm1* conditional knock-out mice, harboring a ROSA26-Cre^ERT^ recombinase, was induced at P0 and mice were euthanized at P42 to harvest kidneys.

### Human cells and tissues

Sections from ADPKD patients and normal human controls and primary ADPKD cells and NHK (normal human kidney) cells were supplied by the PKD Biomaterials Research Core laboratory as described previously^45^. A protocol for the use of discarded human kidney tissues was approved by the Institutional Review Board (IRB) at the University of Kansas Medical Center. All experiments were performed in accordance with relevant guidelines and regulations of IRB. An informed consent was obtained from all the patients or their legal guardians before obtaining the tissue. ADPKD cells or sections were obtained from patients near or at ESRD with an average age of 52 yrs (range: 45–63 yrs). NHK cells or sections were obtained from cadavers whose kidneys were unsuitable for transplantation either because of abnormalities in vasculature or poor perfusion characteristics. Average age for these individuals was 44 yrs. ADPKD cells were obtained from multiple surface cysts ranging in size. NHK cells were cultured from sections of cortex. These cells have shown to be positive for collecting duct marker, *Dolichos biflorus agglutinin* (DBA)^8^. Cells were cultured in DMEM/F-12 supplemented with 5% FBS, 5 µg/ml insulin, 5 µg/ml transferrin, and 5 ng/ml sodium selenite (ITS, Thermo Scientific) and penicillin (100 U/ml), streptomycin (130 µg/ml) (Pen/Strep).

### Antibodies and reagents

Antibodies against N3 (for Western blots), J1, J2, N2, and N4 were from Abcam (Cambridge, MA). Antibodies against Dll1, and Dll4 were from LifeSpan Biosciences (Seattle, WA), antibodies for N1 (val1744), Creb1, AKT and Dll3 were from Cell Signaling Technology (Danvers, MA). Anti-PCNA anti- B actin antibodies were from Sigma Aldrich (St. Louis, MO). Anti-Hes, anti N3 (for IHC) and anti Lamin B1 antibodies were purchased from Santa Cruz (Santa Cruz Biotechnology, Santa Cruz, CA). Anti-β-actin antibody was purchased from Thermo-Scientific (Freemont, CA). Fluorescein labeled *lotus tetragonolobus* (LTA) and fluorescein labeled *dolichos biflorus agglutinin* (DBA) were from Vector Laboratories (Burlingame, CA). Gamma secretase inhibitors DAPT [*N*-(*N*-(3,5-difluorophenacetyl)-l-alanyl)-*S*-phenylglycine *t*-butyl ester], Dibenzazepine (DBZ) were purchased from ApexBio (Houston, TX) and PF3084014 (Nirogacestat) was from BOC Sciences, (Shirley, NY).

### Cell culture and treatments

To evaluate Notch expression, NHK cells and ADPKD cells were grown to 80% confluency in 10 mm cell culture dish. Cells were washed and lysed with RIPA lysis buffer (50 mM Tris HCl pH7.5, 137 mM NaCl,1% IGEPAL, 2 mM EDTA, and Complete Protease Inhibitor (Thermo scientific)) for Western blots. For treatments, ADPKD cells were grown to 70% confluency followed by 24-hour low serum (0.001% and no ITS) treatment. Cells were then treated with vehicle or (DMSO) or DAPT (50 µM), for 15 minutes before adding 5 µM FSK and/or 5 mg/ml EGF. These treatments were done in regular growth media. For ERK measurements, cells were harvested after 15 minutes of treatment, while for N3 and PCNA measurements after 24 hours of treatment.

### Western blots

Cells were washed with PBS three times and lysed with 500 μl of RIPA lysis buffer. Fresh or frozen kidney tissues were chopped into small pieces and lysed in RIPA buffer with protease inhibitors using 50–80 strokes with a dounce homogenizer. Nuclear and cytoplasmic extractions were done using nuclear extrcation kit (Abcam). Protein was measured using BCA protein assay (Bio-Rad, Hercules, CA). Whole cell lysates (50 to 100 μg) were solubilized in 4xNuPage (Novex) sample buffer (containing 25% tris(2-carboxyethyl) phosphine (TCEP)) heated to 65 °C for ten minutes and electrophoresed on 10% polyacrylamide gels. Proteins were transferred to PVDF membranes. The immunoblots were blocked in 5% nonfat dry milk in PBST (PBS containing 0.1% Tween 20) for 1 hour at room temperature and then followed by PBS washes; the blots were incubated with appropriate dilutions of primary antibodies overnight. The blots were then washed three times at room temperature with PBST and incubated with secondary antibodies (1:10,000 dilution in blocking solution) for 1 hour at room temperature. After three washes in PBST, bound antibody was detected by chemiluminescence (Western Lightning Plus ECL, Perkin Elmer).

### Immunohistochemistry (IHC)/Immunofluorscence (IF)

IHC was performed as described previously^12^. Briefly, kidney sections from wild type and PKD mice were deparaffinized with Xylene and hydrated with graded ethanols. Sections were then boiled in citrate buffer (10 mM sodium citrate, 0.05% tween 20, pH: 6.0) and cooled to room temperature. Sections were incubated for 30 min with 3% hydrogen peroxide for IHC and 0.5 M ammonium chloride for IF to block endogenous peroxidase/fluorescence activity. Subsequent washing in PBS and blocking with 10% normal serum (in PBS from the species the secondary antibody was raised in) for 1 h were followed by incubation for 1 h with primary antibodies in a humidified chamber. Slides were washed three times in PBS and incubated for 1 h in 1:400 diluted biotin-conjugated secondary antibodies (Vector Laboratories, Burlingame, CA) for IHC and fluorescein/Texas red-conjugated antibodies for IF. Slides were washed four times in PBS for 5 minutes each. For IF, the slides were coverslipped using vecta shield (Vector Labs). For IHC, the slides were further incubated with avidin-biotin-peroxidase complex (ABC Elite; Vector Laboratories, Burlingame, CA) and detected with diaminobenzidine (DAB; Sigma Aldrich, St. Louis, MO). Tissue sections were then dehydrated with graded ethanols and mounted with Permount (Fisher Scientific, Pittsburg, PA). Slides were viewed on a Leica DMR microscope equipped with an Optronics Magnafire digital camera.

### Immunocytochemistry (ICC)

Immunocytochemistry was performed in NHK and ADPKD cells that were grown in 8-well chamber slides to 80% confluency. The cells were fixed in 4% paraformaldehyde for fifteen minutes followed by incubation with100 mM ammonium chloride for five minutes twice. Cells were then permeabilized with 0.05% Triton in PBS for five minutes. The slides were blocked with 10% BSA for 1 hr. Primary antibody and secondary antibody steps were same as described above for IFF. After mounting with Vectashield containing DAPI (Vector Laboratories), slides were analyzed under confocal microsopy or a Leica DMR microscope.

### *In vitro* cyst formation assays and Notch inhibition

*In vitro* cyst assays were performed as described previously^46^. Briefly, primary cultures of ADPKD cells were suspended in media containing cold Type 1 collagen (PureCol) in a 96-well plate. The media was formulated to contain the following contents per 15 ml: 5.41 ml 2.780x Minimum essential medium (MEM) (Invitrogen), 0.15 ml 1 M HEPES, 0.75 ml 5% sodium bicarbonate, 0.123 ml 1 N Sodium hydroxide, 7.84 ml cold collagen, 0 0.75 ml cells (4 × 10^3^ per well per 100ul for ADPKD cells). Immediately after adding collagen and cells, 100ul of this media was pipetted into each well of 96 well plate. The plate was allowed to incubate at 37 °C for 45 minute so that the collagen would polymerize with cells suspended throughout the gel. Then, 150 μl of defined media (1:1 DMEM/F12 with ITS, 5 × 10^−8^ M hydrocortisone, 5 × 10^−5^ M triiodothyronine) was added either alone to serve as control or containing 5 μM forskolin and 5 ng/ml EGF to initiate cyst growth. Following cyst growth between day 5 to 6, the agonists (FSK and EGF) were removed and the gels were rinsed twice with defined media. To initiate Notch inhibition, control media or media containing 50 µM DAPT (N-(N-(3,5-Difluorophenacetyl)-S phenylglycine t-butyl ester), 1–2 µg/ml DBZ (Dibenzazepine) or 2–5 nm PF3084014 (PF) was added and replaced with fresh treatment every day. After 5–7 days, the outer diameter of cross sectional images of spherical cysts with distinct lumens were measured using digital camera attached to an inverted microscope and analysed with video analysis software. Surface area was calculated from the outer diameters and total surface area of the cysts was determined from the sum of individual cysts within each well. Cysts with diameters of 50 μm or less were excluded. Data is presented as surface area/cm^2^ or surface area fold change to account for patient to patient variation. Experiments were replicated six times from at least three different ADPKD patients.

### Statistics

Data are expressed as mean ± SE. Statistical significance was measured by Student’s unpaired T test for comparison between control and PKD groups. One way Anova was performed to compare more than two groups followed by Tukey HSD test. A P value of <0.05 was considered statistically significant.

## Electronic supplementary material

Supplementary Information
