# Supplementary material for: Aberrant Regulation of Notch3 Signaling Pathway in Polycystic Kidney Disease
Source: Sci Rep. 2018 Feb 20;8:3340. doi: 10.1038/s41598-018-21132-3 (PMC5820265; doi:10.1038/s41598-018-21132-3)

Aberrant Regulation of Notch 3 Signaling Pathway in Polycystic Kidney Disease.

Jessica Idowu [1, 5], Trisha Home [1, 5], Nisha Patel [1, 5], Brenda Magenheimer [2, 5], Pamela V Tran [3, 5], Robin L Maser [4,5], Christopher J Ward[1, 5], James P. Calvet [2, 5], Darren P Wallace[1, 5] and * Madhulika Sharma[1,5]

Departments of [1]Internal Medicine, [2]Biochemistry and Molecular Biology, [3]Anatomy and Cell Biology, [4]Clinical Laboratory Sciences and [5]The Jared Grantham Kidney Institute, University of Kansas Medical Center, Kansas City, Kansas, United States

Correspondence to:

Madhulika Sharma, PhD

Department of Internal Medicine

University of Kansas Medical Center

Kansas City, KS 66160

Email: msharma3@kumc.edu

Phone: 913 945 9393 (office)

Fax: 913 588 9252

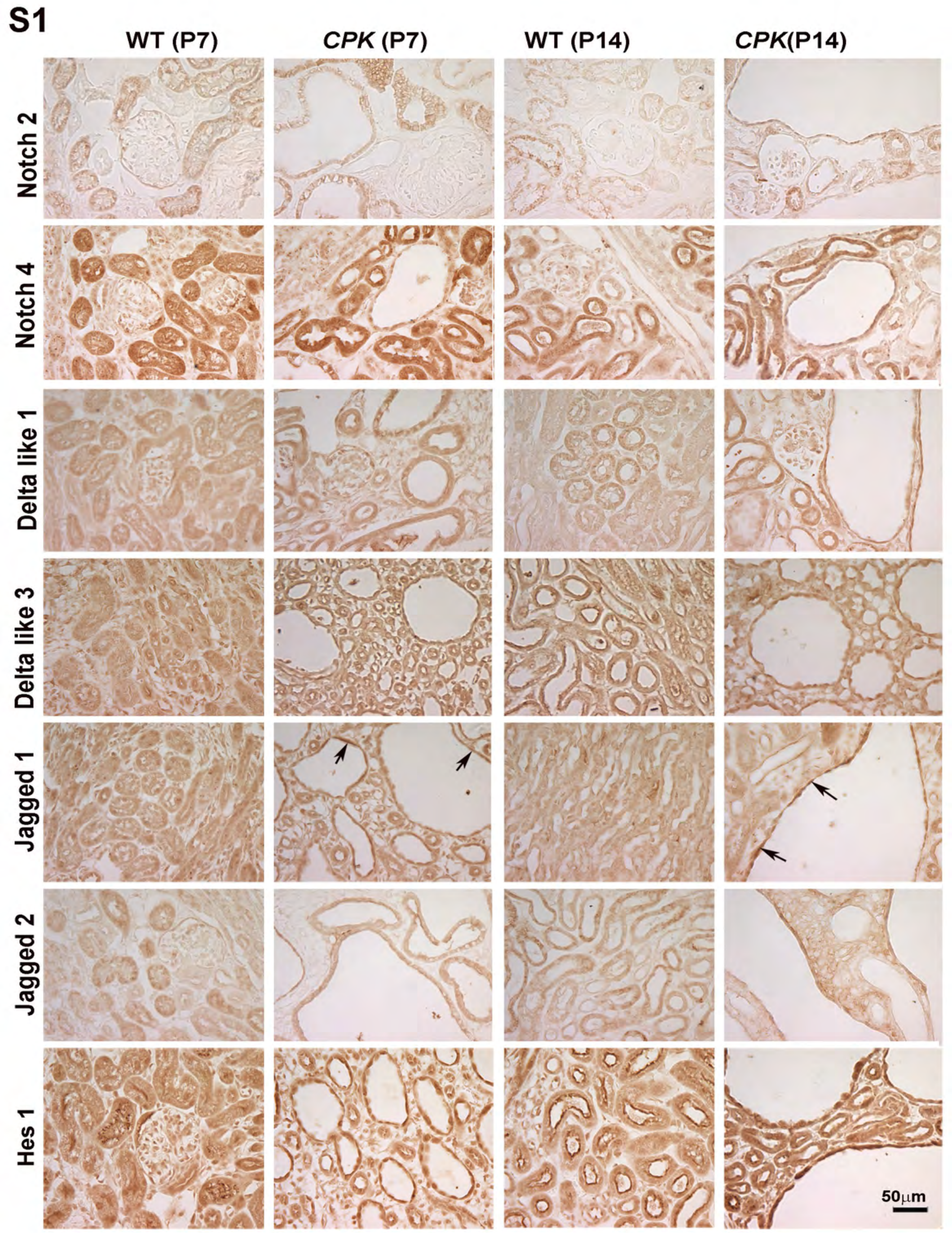
S1
WT (P7)
CPK (P7)
WT (P14)
CPK(P14)
Notch 2
Notch 4
Delta like 1
Delta like 3
Jagged 1
Jagged 2
Hes 1
50μm

S2

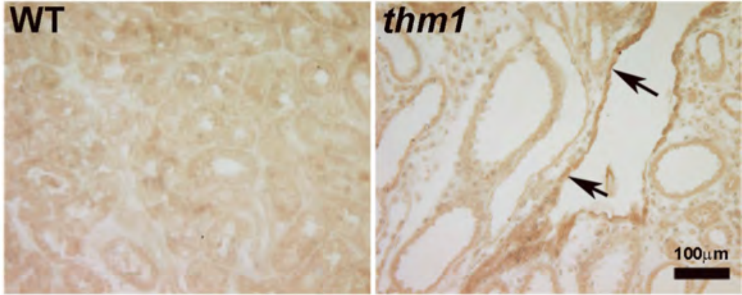

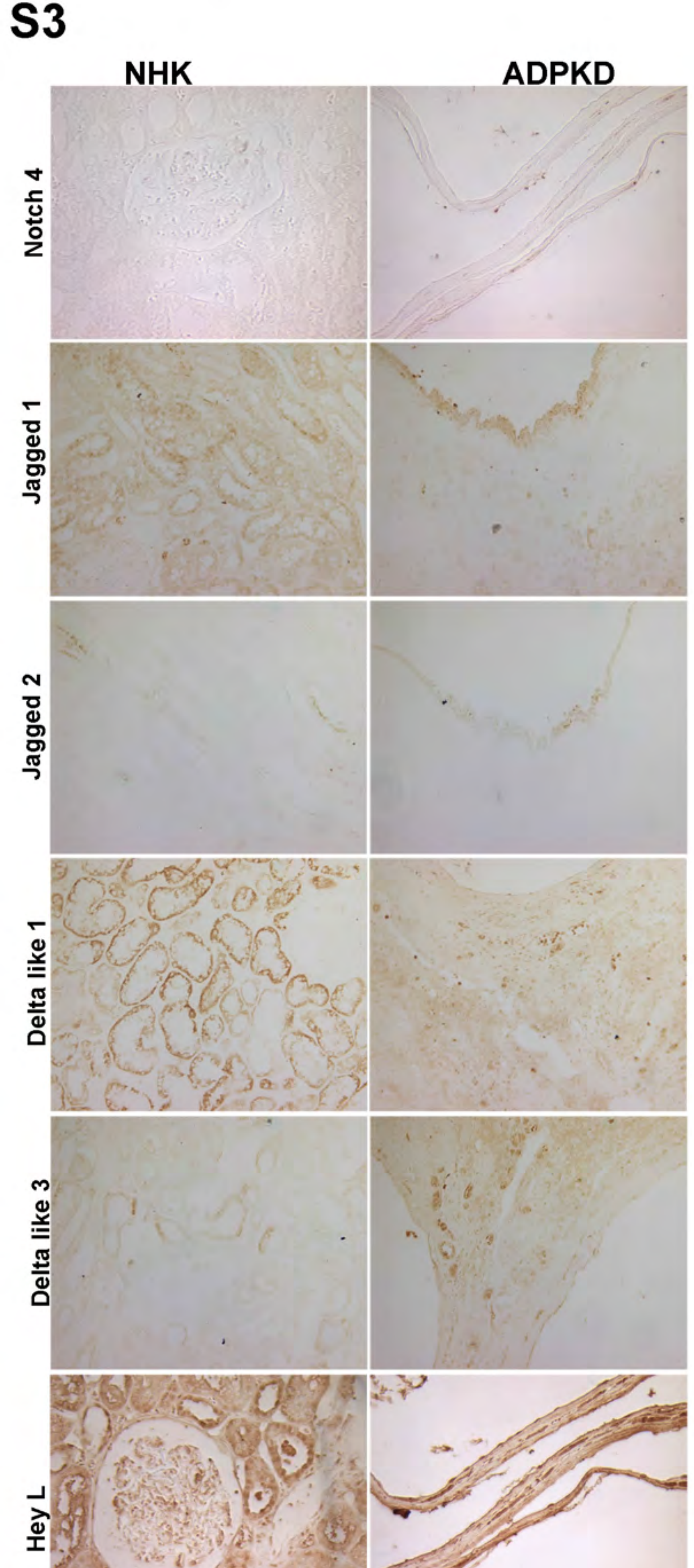
S3
NHK
ADPKD
Notch 4
Jagged 1
Jagged 2
Delta like 1
Delta like 3
Hey L
50μm

S4

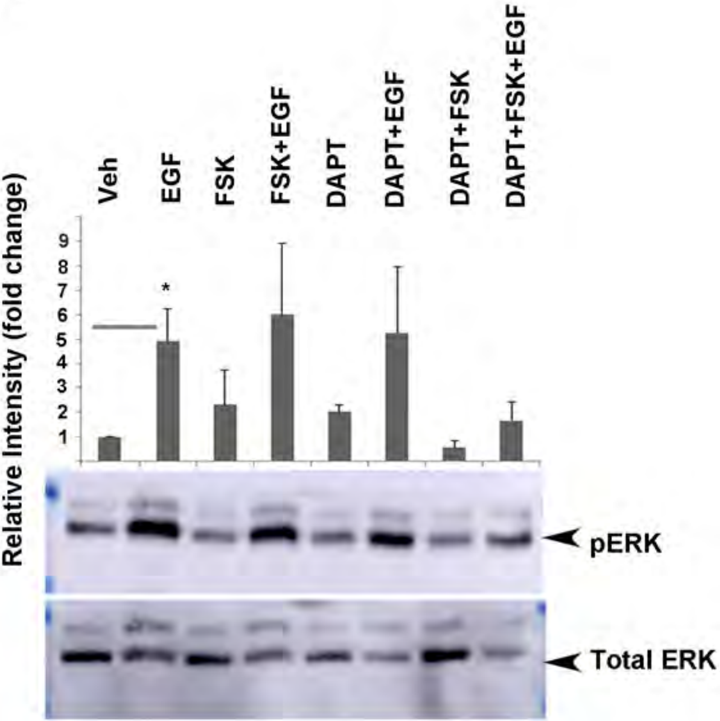

Veh
EGF
FSK
FSK+EGF
DAPT
DAPT+EGF
DAPT+FSK
DAPT+FSK+EGF
Relative Intensity (fold change)
9
8
7
6
5
4
3
2
1
*
pERK
Total ERK

S5

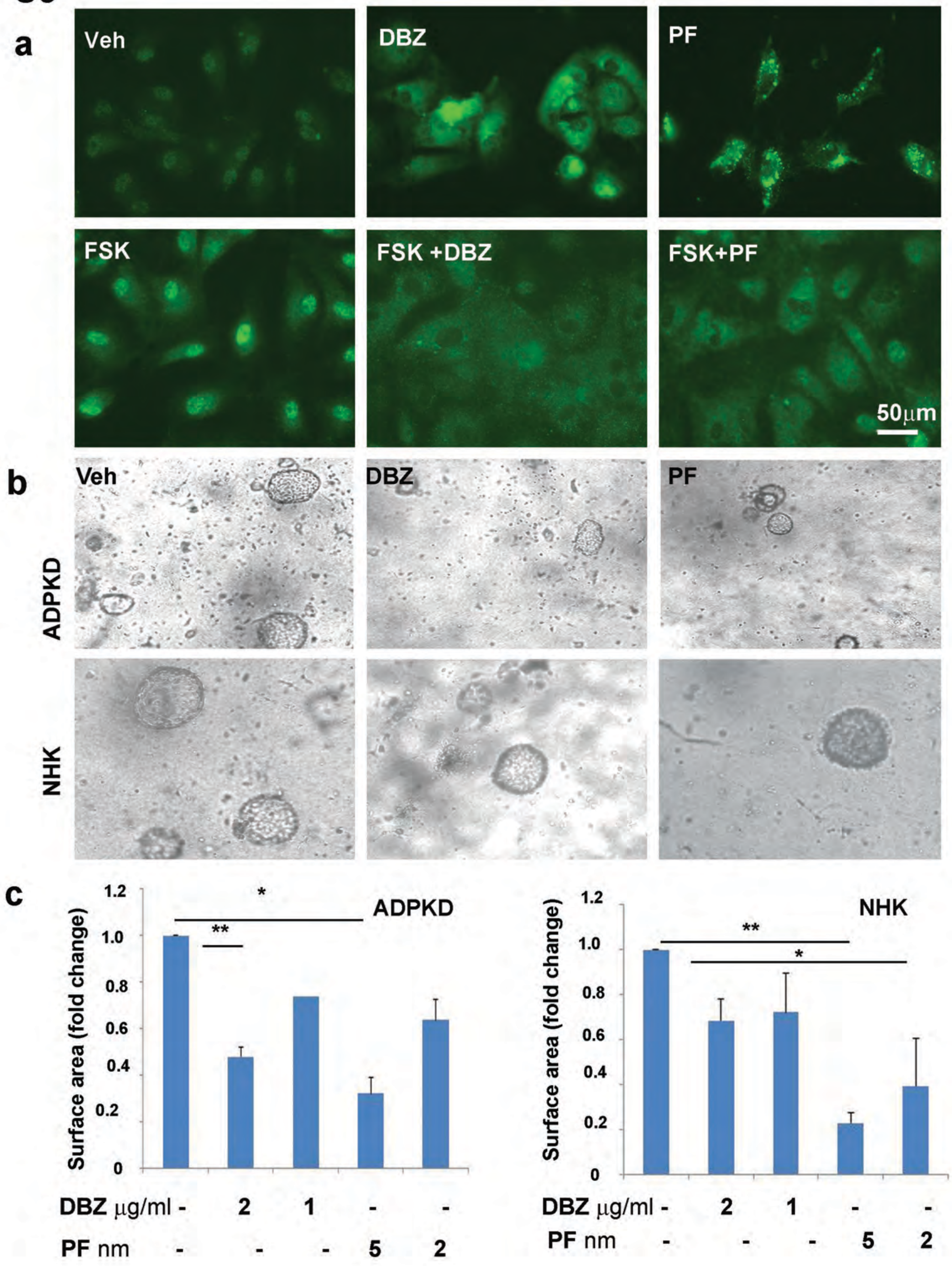
a
Veh
DBZ
PF
FSK
FSK +DBZ
FSK+PF
50μm
b
Veh
DBZ
PF
ADPKD
NHK
c
Surface area (fold change)
ADPKD
NHK
1.2
1.0
0.8
0.6
0.4
0.2
0
*
**
DBZ μg/ml - 2 1 - -
PF nm - - - 5 2

Supplementary figure legends:

Figure S1. Expression pattern of Notch pathway members in kidneys from ARPKD mouse model: IHC for Notch pathway components on P7 and P14 WT and *cpk* kidneys. Arrows show increased staining for Jagged1 in cyst-lining epithelial cells of *cpk* kidneys. Data are representative of 3 WT and 3 *cpk* mice.

Figure S2. Increased expression of Notch3 in renal cystic epithelial cells of ciliary mutant model of PKD: IHC for N3 on kidney sections of P42 WT and *Thm1* conditional knock-out mice reveals increased Notch3 in cyst-lining epithelial cells, indicated by arrow. Data are representative of 3 WT and 3 *Thm1* cko mice.

Figure S3. Expression pattern of Notch pathway members in human NHK and ADPKD kidneys: IHC for Notch pathway members on NHK and ADPKD kidney sections. Data are representative of 4 NHK and 4 ADPKD patient samples.

Figure S4. Response of Notch inhibition on ERK activation: (a) Western Blots were performed for phospho-ERK and ERK to evaluate ERK activation after 15-minute treatment of ADPKD cells. P-ERK/ERK ratios were quantified and shown as fold-change. Experiments were performed three times using three different ADPKD cell lines. Data are represented as mean ± standard error. *$P < 0.05$

Figure S5. Effects of gamma secretase inhibitors on Notch3 expression and cyst formation. (a) ADPKD cells were grown to 70% confluency, then treated with FSK, vehicle (Veh), 2µg/ml Dibenzazepine (DBZ), 5nm PF3084014 (PF) or both DBZ and PF for 24 hrs. Immunocytochemistry was performed to detect N3 expression. **(b)** ADPKD/NHK cells were grown in collagen gels with cyst agonists, FSK and EGF. After 6 days when cysts were formed, agonists were removed and cells were treated with vehicle (Veh), 1-2µg/ml Dibenzazepine (DBZ) or 2-5nm PF3084014 (PF) for six more consecutive days. Shown are representative images of cysts after treatment. **(c)** Cyst diameters were measured and converted to surface area. Shown are differences in cyst surface area (fold change) by treatment (n=3). Treatment with agonists in the absence of GSI's was expressed as 1.0. $^{**}P < 0.01$, $^{*}P < 0.05$.

Figure 1 full blots (original exposure)

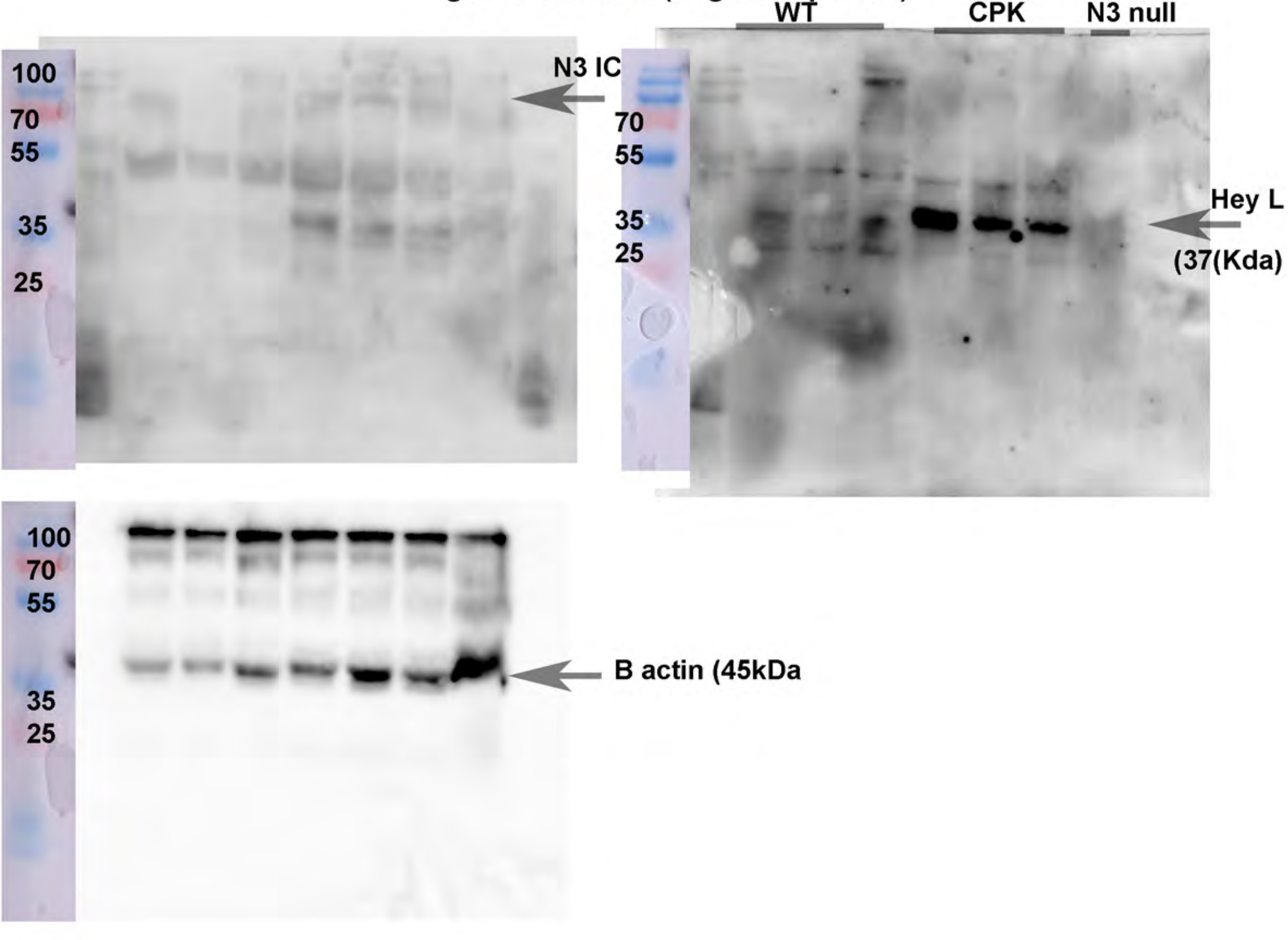

Full blots for figure 2(original exposure)

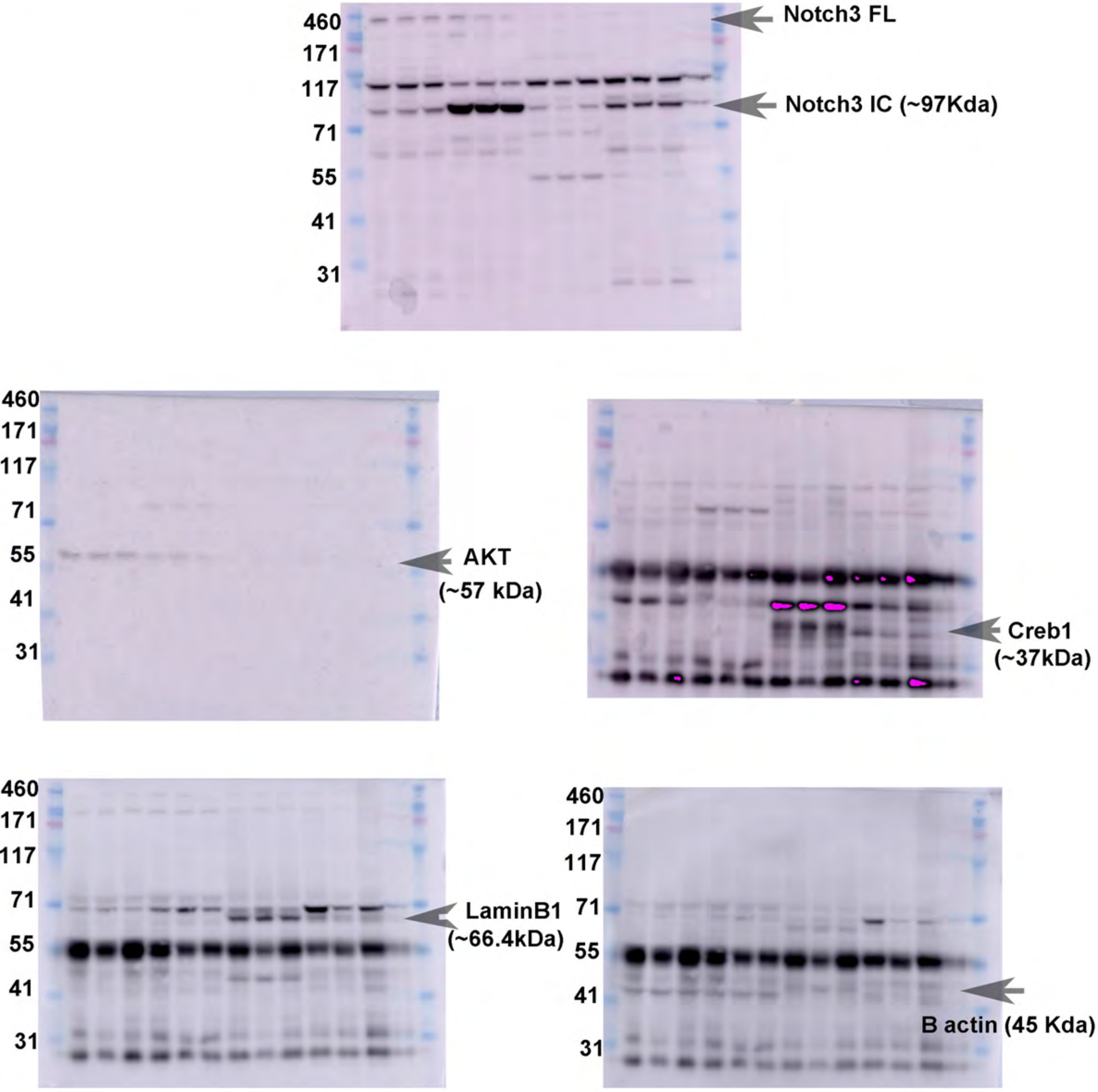

Figure 3 full blots (original exposure)

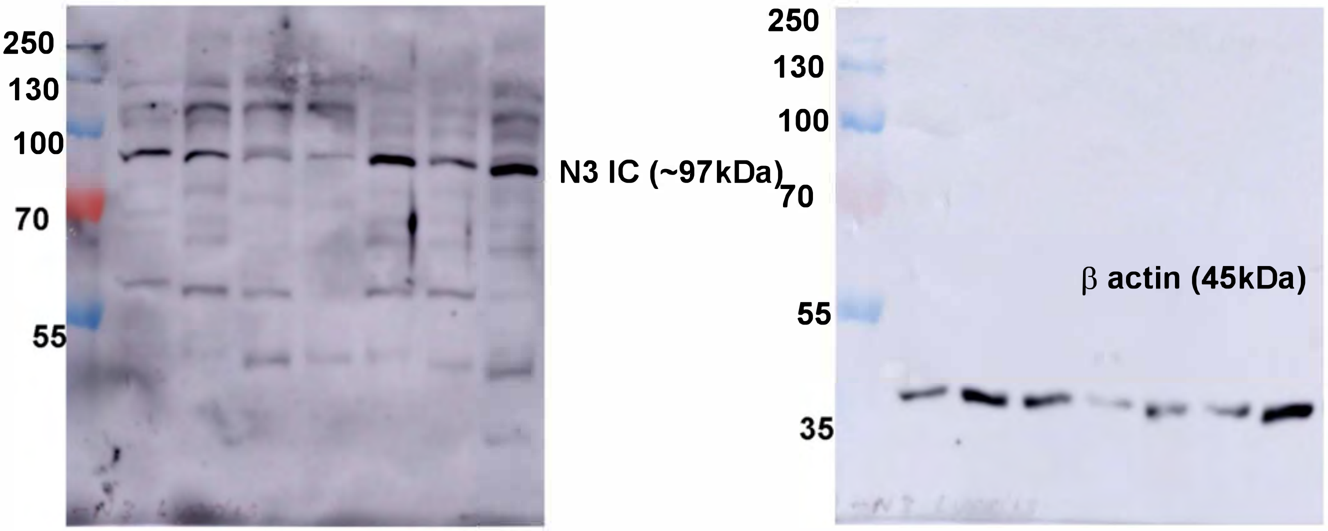

Figure 5 full blots

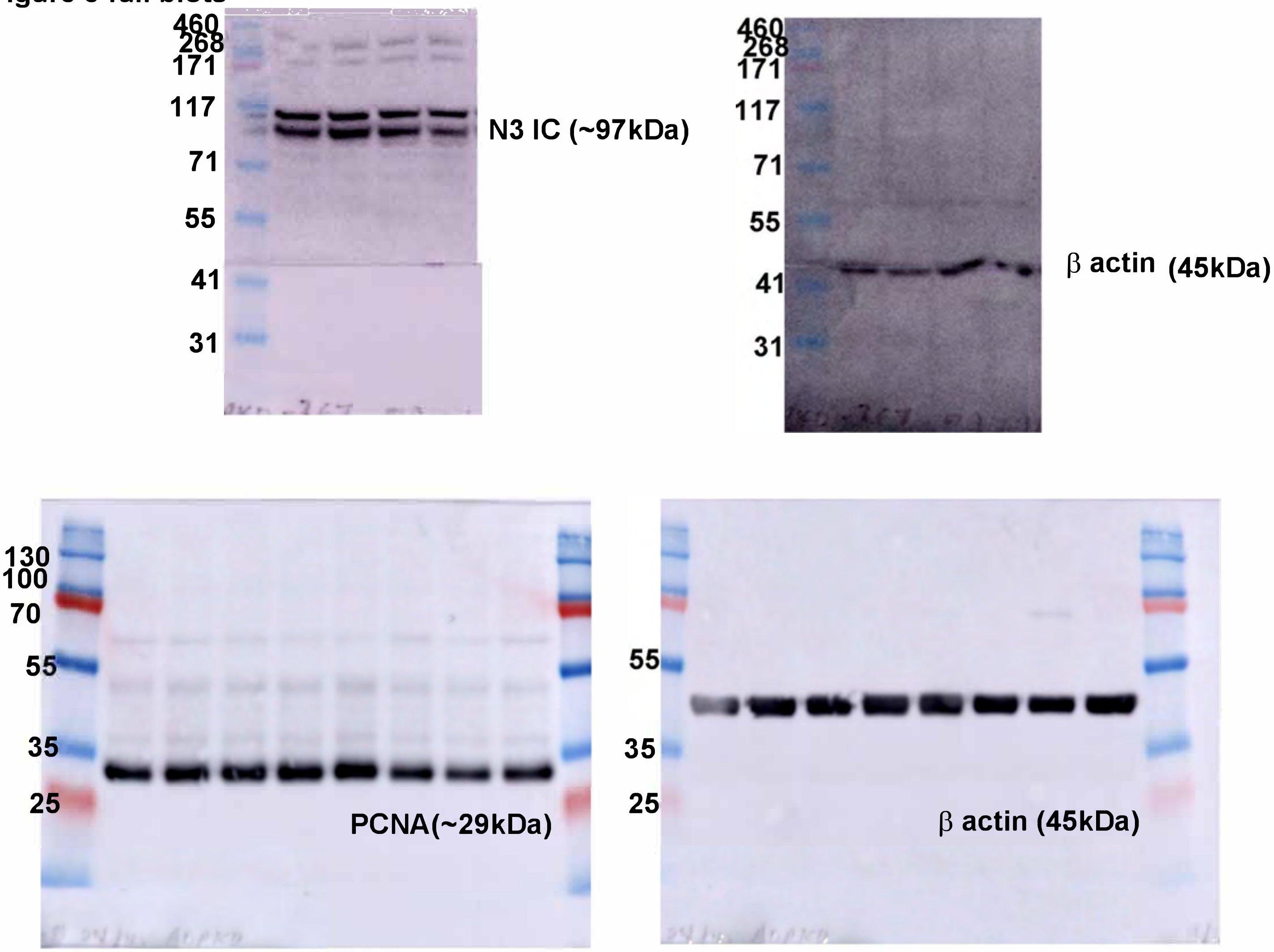

Supplement: Supplementary file 1 — Supplementary Information [file 41598_2018_21132_MOESM1_ESM.pdf]
